# Haematopoietic Stem Cell Transplantation Results in Extensive Remodelling of the Clonal T Cell Repertoire in Multiple Sclerosis

**DOI:** 10.3389/fimmu.2022.798300

**Published:** 2022-02-07

**Authors:** Jennifer Massey, Katherine Jackson, Mandeep Singh, Brendan Hughes, Barbara Withers, Carole Ford, Melissa Khoo, Kevin Hendrawan, John Zaunders, Bénédicte Charmeteau-De Muylder, Rémi Cheynier, Fabio Luciani, David Ma, John Moore, Ian Sutton

**Affiliations:** ^1^ Department of Haematology, St Vincent’s Hospital, Darlinghurst, NSW, Australia; ^2^ Department of Neurology, St Vincent’s Hospital, Darlinghurst, NSW, Australia; ^3^ Blood Stem Cell and Cancer Research Group, St Vincent’s Centre for Applied Medical Research, Darlinghurst, NSW, Australia; ^4^ St. Vincent’s Clinical School, Faculty of Medicine, University of New South Wales (UNSW), Darlinghurst, NSW, Australia; ^5^ Immunogenomics Lab, Garvan Institute of Medical Research, Darlinghurst, NSW, Australia; ^6^ School of Medical Sciences and Kirby Institute for Infection and Immunity, University of New South Wales (UNSW), Kensington, NSW, Australia; ^7^ Immunology Laboratory, St Vincent’s Centre for Applied Medical Research, Darlinghurst, NSW, Australia; ^8^ Université de Paris, INSERM, CNRS, Institut Cochin, Paris, France; ^9^ Department of Neurology, St Vincent’s Clinic, Darlinghurst, NSW, Australia

**Keywords:** multiple sclerosis, aHSCT, T cell, TCR - T cell receptor, public clonotypes

## Abstract

Autologous haematopoietic stem cell transplantation (AHSCT) is a vital therapeutic option for patients with highly active multiple sclerosis (MS). Rates of remission suggest AHSCT is the most effective form of immunotherapy in controlling the disease. Despite an evolving understanding of the biology of immune reconstitution following AHSCT, the mechanism by which AHSCT enables sustained disease remission beyond the period of lymphopenia remains to be elucidated. Auto-reactive T cells are considered central to MS pathogenesis. Here, we analyse T cell reconstitution for 36 months following AHSCT in a cohort of highly active MS patients. Through longitudinal analysis of sorted naïve and memory T cell clones, we establish that AHSCT induces profound changes in the dominant T cell landscape of both CD4+ and CD8+ memory T cell clones. Lymphopenia induced homeostatic proliferation is followed by clonal attrition; with only 19% of dominant CD4 (p <0.025) and 13% of dominant CD8 (p <0.005) clones from the pre-transplant repertoire detected at 36 months. Recovery of a thymically-derived CD4 naïve T cell repertoire occurs at 12 months and is ongoing at 36 months, however diversity of the naïve populations is not increased from baseline suggesting the principal mechanism of durable remission from MS after AHSCT relates to depletion of putative auto-reactive clones. In a cohort of MS patients expressing the MS risk allele HLA DRB1*15:01, public clones are probed as potential biomarkers of disease. AHSCT appears to induce sustained periods of disease remission with dynamic changes in the clonal T cell repertoire out to 36 months post-transplant.

## Introduction

Multiple sclerosis (MS) is an inflammatory condition of the central nervous system (CNS) that is presumed to be mediated by an aberrant adaptive immune response (1). CSF-specific oligoclonal IgG is detected in the majority of affected individuals (2) and the MHC class II allele HLA-DRB1*15:01 has been demonstrated to confer a three-fold increase in the risk of MS susceptibility in numerous gene association studies across diverse population groups (3–5). T-cell receptor (TCR) analysis of MS lesions *post-mortem* has identified clones across anatomically distinct regions with “silent” nucleotide exchanges (different nucleotides coding for the same amino acid) within the V-CDR3-J region (6), supporting the concept of T-cell recruitment to a common antigenic driver of disease within the CNS. A paradigm shift in MS treatment, in favor of earlier high-efficacy therapy (7–9), along with increased recognition of the complications of long-term immune suppression in MS (10, 11) has led to interest in immuno-ablative therapies (7), including autologous haematopoietic stem cell transplantation (AHSCT). AHSCT is associated with periods of disease-free remission extending well beyond the period of immune reconstitution (IR) (12). The future development of targeted IR therapies that lack the toxicity of chemotherapy necessitates an improved understanding of disease pathogenesis.

Highly shared TCR amino acid sequences are involved in public T cell responses (identical clonotypes identified across individuals) to acute and persistent pathogens, but also auto- and allo-reactive immune responses (13–17). Given the extensive nature of public TCRs, it is suggested that they arise from a fundamental feature of T cell biology whereby convergent recombination readily facilitates the production of certain amino acid sequences and enables the sharing of TCRs across individuals (18). Recent studies have investigated the clonal T lymphocyte repertoire of MS patients utilising high throughput TCR sequencing (19–22). Shared T-cell clones have been detected at low frequencies in blood, CSF and the CNS within individual patients, however dominant clones appear unique to each compartment. Although public TCRs across multiple MS patients have been published (19–22) their pathogenicity versus a stochastic requires further investigation.

While defining public pathogenic T-cell clones in MS has to date proved elusive, a consistent pattern of immuno-ablation and immune reconstitution of T-cell populations following AHSCT has been established (23–32). Following administration of an immuno-ablative conditioning regimen there is a bimodal recovery of T-cell populations. Early expansion of predominately CD8+ memory T-cells, which proliferate readily in the lymphopenic environment and may eventually acquire an anergic phenotype, is followed by a slower recovery of naïve thymically-derived T lymphocytes (24, 26–28). Two publications have assessed the TCR repertoire following AHSCT for MS. Earlier work by Muraro *et al.* reported that at 24 months post-AHSCT the CD8+ lymphocyte pool is dominated by an expansion of pre-existing T-cell clones, whilst the CD4+ pool is primarily comprised of clones not present pre-transplant (25). However, in this study, cells were not sorted beyond CD4+ and CD8+ subsets with no analysis of naïve or memory status. Furthermore, older repertoire sequencing studies suffered from high rates of primer bias which has been overcome with newer UMI-based technology (33). In a separate publication, TCR repertoires of naïve and memory CD4+ and CD8+ subsets pre- and 24-months post-AHSCT showed changes in heatmap profiles of TCR clonality between baseline and follow-up (34). Neither study serially surveyed the TCR repertoire at multiple time points up in order to define the clonal kinetics of immune reconstitution as it relates to MS.

Thymic production of T-cells is the sole path by which humans generate T-cell repertoire diversity (35–38). Diversity of the TCR repertoire occurs through random receptor generation (VDJ recombination) and T cell selection, determined by recognition of self-peptide. Novel software tools can model the generation and selection of the TCRβ chain, quantifying the variability of the two processes in a population (39) and an individual (40). These modelling algorithms have the potential to scrutinize TCR repertoires of patients with autoimmune disease for autoreactive clones. Recently, high-dimensional TCR sequencing analysis has assessed clusters of clones in the CSF and peripheral blood of MS patients, confirming the effect of MS on the TCR clonal profile (41). MS is presumed to arise as a result of auto-reactive T-cells (42), which are likely to escape negative selection in the thymus and then become activated following interaction with undefined environmental factors. Therefore, it is plausible that AHSCT results in ablation of pathogenic clones, which are not regenerated in the naïve repertoire.

In this study we applied high-throughput repertoire sequencing and TCR profiling to sorted populations of naïve (CD45RA+) and memory (CD45RO+) CD4+ and CD8+ lymphocytes derived from MS patients followed longitudinally out to 36 months post-AHSCT in order to investigate the clonal kinetics of immune reconstitution. Through the analysis of TCRβ mRNA from sorted naïve and memory clones we aimed to characterise the effect of AHSCT on T cell repertoires, identify public clonotypes with characteristics of potential auto-reactivity and to assess the role of thymopoiesis in relation to MS susceptibility.

## Materials and Methods

### Study Design

Samples were derived from individuals entered into a phase 2 clinical trial of AHSCT for MS commenced in December 2010 (amended 2013, 2015, 2018) (HREC Ref: HREC/10/SVH/135) that was registered with the Australian New Zealand Clinical Trials registry (ACTRN12613000339752). All patients gave informed consent for tissue banking. A scheduled interim analysis, performed in December 2017, assessed the outcomes of patients with more than 12 months follow-up. Eligibility criteria and clinical trial end points have been previously published (43). Relapses are defined as *(1)* clinical relapse, defined by the patient’s neurologist and requiring treatment with steroids; *(2)* new/enlarging T2 lesions and/or new gadolinium enhancing lesions on centralised MRI review following re-baseline MRI scan at 6 months.

This study is an exploratory cohort study utilising bio-specimens from the first 35 patients in the clinical trial. This initial cohort included both relapsing-remitting and secondary progressive (SP) MS, however the trial protocol was later updated to exclude SPMS patients due to poor efficacy. SPMS patients (n=15) were therefore excluded from this immunological analysis since disability progression in MS is a multifactorial process distinct from the causes of inflammatory disease activity (44). A further patient (n=1) was censored due to restarting immunotherapy. Of the 19 remaining patients incorporated in the immunophenotyping analysis, 13 had adequate number of PBMCs cryopreserved to enable TCR analysis and underwent T cell repertoire sequencing. A cohort of comparator patients with MS treated with high-efficacy disease modifying therapy (natalizumab) also underwent biobanking as part of the same clinical trial. Two 10mL EDTA peripheral blood specimens were obtained from patients with written consent after Human Research Ethics Committee approval. All patient samples were de-identified using a laboratory 10 digit ID code. Laboratory analysis was performed blinded to the clinical outcome.

### Transplant Procedure

Stem cell mobilisation was undertaken using a single dose of cyclophosphamide (Cyc) 2g/m^2^ intra-venously and granulocyte-colony stimulating factor (G-CSF) 10mcg/kg per day subcutaneously for 10 days with appropriate supportive care. Peripheral blood stem cells were collected by leukoapheresis using a Cobe Spectra (Terumo BCT, Colarado, USA), and cryopreserved. Transplant conditioning was performed with BEAM chemotherapy: carmustine 300mg/m^2^, on day -6; etoposide 200mg/m^2^ and cytarabine 200mg/m^2^, on day -5 to -2; and melphalan 140mg/m2, on day -1. On day 0, the unmanipulated leukapheresis product containing CD34+ cells was thawed and infused unmanipulated. Horse anti-thymocyte globulin (ATG) 20mg/kg/day was administered on day +1 and +2.

### HLA Typing

HLA typing was performed by the Australian Red Cross Blood Service. HLA typing was low resolution for class I loci and resolved to 4 digits at the class II loci.

### Peripheral Blood Mononuclear Cells

Patient peripheral blood samples were collected with informed consent at pre-specified time points during clinical evaluation [as published (43)]. A cohort of comparator patients with MS treated with high-efficacy disease modifying therapy (natalizumab) also underwent biobanking as part of the same clinical trial. Two 10mL EDTA peripheral blood specimens were obtained from patients with written consent after Human Research Ethics Committee approval. Peripheral blood mononuclear cells were separated on a Ficoll-Paque density gradient and cryopreserved (10% dimethylsulfoxide) for further use. All experiments were performed with cell viability > 90%.

### Flow Cytometric Analysis of Immune Reconstitution

Antibodies and compensation beads used have been tabulated (**Supplementary Tables 1**, **2**). In brief; thawed PBMCs were resuspended in 1mL of PBS. Viability was assessed with the addition of 1μL of FVS700 and the solution was incubated at 37°C for 10 minutes. Viability dye was then blocked with 1mL of heat inactivated FBS. The sample then underwent two wash cycles at 300g with PBS, and the sample was subsequently resuspended at a ratio of 10^6^/100μL. Cells were stained with the antibodies at the dilutions listed below. 150μL of 0.5% PFA was added to the samples prior to storage in the dark, at 4°C. Sample acquisition was performed on a LSRFortessa (Becton Dickinson, NJ, USA) within 1 hour of preparation being completed. The populations of interest are outlined in **Supplementary Table 3**. The gating strategy has been outlined in **Supplementary Figure 1**.

### TREC

T-cell receptor Excision Circles (TRECs) were quantified on patient’s PBMCs as described in (45, 46). In brief, approximately 3x10^6^ PBMCs were thawed, washed in DPBS and pelleted. Cells were then lyzed in 10mM Tris-HCl, 0.05% Tween 20, 0.05% NP40 and Proteinase K (100 µg/mL) for 30 minutes at 56°C and then 15 minutes at 98°C. Real-time PCR quantification of the different TRECs was performed using LightCycler technology (Roche Diagnostics). Through PCR amplification, any TREC was coamplified with the CD3γ gene (used as a housekeeper). Specific primers for the sjTRECs (byproducts of the TCRδ locus excision), DJβTRECs (byproducts of TCRBD1/TCRBJ1.1 to TCRBJ1.6 or TCRBD2/TCRBJ2.1 to TCRBJ2.7 rearrangements), and CD3γ gene were defined on human sequences (GenBank accession numbers NG_001333, NC_00007.14, and NG_007566). Plasmids containing any of the TREC amplicon and the CD3γ amplicon were used to generate standard curves.

### FACS

Fluorescence-activated cell sorting (FACS)-sorted T cell sub-populations were acquired for TCR profiling. A multicolour flow cytometry panel was developed consisting of 6 colours to detect four T cell subpopulations (**Supplementary Table 4**). PBMCs were thawed and resuspended in 1mL of PBS. Viability was assessed with 1μL of FVS700 and the suspension was incubated at 37°C for 10 minutes. Viability dye was then blocked with 1mL of heat-inactivated FBS. The sample then underwent two wash cycles at 300g with PBS, and the sample was subsequently resuspended at a ratio of 10^6^/500μL. Cells were stained with the antibodies (**Supplementary Table 5**) at the volumes listed. 2ml of 1x PBA was added to all flow tubes prior to an additional wash, and cells were subsequently resuspended in 500μL of FACS buffer. FACS was performed on a FACS Aria II (BD). The gating strategy has been outlined in **Supplementary Figure 2**.

### RNA Extraction and Storage

RNA extraction was performed using the RNeasy Micro kit. All components were stored as instructed. RNA concentration and RNA quality score (RIN number) were acquired using the ‘pico’ assay of the LabChip GX Nucleic Acid Analyser. RNA was then frozen at -80°C until sequencing.

### TCR RNA Deep Sequencing

TCR high throughput RNA sequencing methods have been previously published (47) and adapted to a local protocol described in brief here.

Reverse transcription of RNA into barcoded cDNA was performed using a modified SmartSeq2 protocol (48) that incorporated a 10bp universal molecular identifiers (UMI) into cDNA molecules to increase the accuracy of measuring the abundance of clones and minimize sequencing errors. Briefly, 2.55μL of RNA was incubated with 1μL dNTPs (10mM) and 1μL oligo-dT primer (10μM) at 72°C followed by addition of an RT mix (100 U SuperScript II reverse transcriptase, 10 U RNAse inhibitor, 1x Superscript II first-strand buffer, 5mM DTT, 1 M Betaine, 6 mM MgCl_2_, 1 μM TSO-UMI primer). The first round of PCR was performed using primers against adapter sequences incorporated during cDNA synthesis with 1x KAPA HiFI HotStart ReadyMix and 8.3 mM Fwd and Rev primer with the following conditions: 98°C for 3 min; [98°C for 20 s, 67°C for 15 s, 72°C for 6 min] x 10 cycles; 72°C for 5 min. PCR products were purified using AMPure XP beads (Agencourt) followed by a second PCR targeting the TCRβ chain. This PCR was performed with 1/8^th^ of the total volume of amplified cDNA with 1x KAPA HiFI Ready Mix and 50mM Fwd and TCRβ primer and the following conditions: 98°C for 45 s; [98°C for 15 s, 60°C for 30 s, 72°C for 30 s] x 30 cycles; 72°C for 1 min. PCR products were purified with AMPure beads followed by barcoding using the Nextera Index kit to enable pooling of multiple samples and sequenced on an Illumina MiSeq at 300bp paired-end reads to a depth of ~0.5 million read pairs per sample.

Primer sequences:

Oligo-dT: AAGCAGTGGTATCAACGCAGAGTACT30VN
TSO-UMI: TCGTCGGCAGCGTCAGATGTGTATAAGAGACAGNNNNNNNNNNACATrGrG+G
Fwd: TCGTCGGCAGCGTCAGATGTGTATAAGAGACAG
Rev: AAGCAGTGGTATCAACGCAGAGT
TCRβ: GTCTCGTGGGCTCGGAGATGTGTATAAGAGACAGTGCTTCTGATGGCTCAAACAC

### Longitudinal T-Cell Clonotype Tracking: Sequencing the Repertoire and Bioinformatics Analysis

Biological replicates; i.e. simultaneous TCR sequencing of two vials of PBMCs from the same subject was performed three times as part of optimisation to ensure sequencing methodology enabled accurate detection of dominant clonotypes. >99% stability was present between biological replicates of the CD45RO+ repertoire, 30-35% stability was present in biological replicates of the CD45RA+ repertoire. TCR repertoire analysis was performed on patients undergoing AHSCT, and patients treated with natalizumab to serve as a treatment comparator. Public clones were defined by their CDR3 AA sequences along with V and J genes. Only clones detected at a size of at least two reads, in two or more individuals were included in the analysis of public clones (**Supplementary Figure 3**).

For estimating the diversity of the TCR repertoire, the Shannon Entropy calculation (49) was used. Clonal expansions, retractions and tracking across samples was evaluated using VDJtools (50). VDJviz (51) was used to assess the relative TCR usage frequency of each Vβ family in CDR3 junction. Additional analysis of TRBV was performed with VDJtools and the tcR package (52). The VDJdb database (53, 54) was screened for known public TCR sequences specific for pathogen antigens, restricted to matched V gene, J gene and CDR3 amino acid sequence where the recorded HLA allele matched the donor MHC haplotype.

GLIPH2 analysis (55) was employed to predict clusters within the datasets defined by shared ‘local similarity’ (patterns/motifs) of the TCR repertoire between samples. GLIPH2 analysis identifies motifs of amino acids within the CDR3 region that are statistically over-represented in one sample compared to another. Analysis was performed using the local OSX binary obtained from http://50.255.35.37:8080/ with default parameters for comparing the AHSCT donor repertoires at each timepoint to the GLIPH2 human reference v2 CD4 or CD8. Results were filtered for Fisher’s score <0.001 (p-value for contingency table comparing motif frequency in sample and reference) and Vβ score <0.05 (whether clusters included biased TRBV usage). To compare TRBV at a single timepoint to baseline, the baseline clonotypes for the 13 AHSCT subjects for CD4+RO+ (or CD8+RO+) were pooled and the necessary reference files were generated with clonotypes, TRBV frequency and CDR3 length. The AHSCT baseline CD4 (or CD8) was then used as the reference dataset to analyse the 6- and 12-month timepoints adjusting minimum CDR3 length to 7 and kmers (AA sequences of k length) from 3 to 7 and results were filtered for Fisher’s score <0.001. SONIA (39, 56, 57) was used to infer thymic selection pressures on features of amino acid CDR3 sequences. Following validation in hundreds of thousands of human αβ TCRs, SONIA takes as input TCR CDR3 AA sequences along with V and J genes. Its output is selection factors for each AA, (relative) position, CDR3 length combinations and V/J gene choice. These selection factors are then used to apply a statistical probability of thymic generation and of thymic selection to each TCR. For each TCR sequence the algorithm computes P_GEN_; the probability of being generated in the process of V-D-J recombination and P_POST_, the probability of entering the peripheral circulation following thymic selection. For determining TCR repertoire overlap across samples, the tcR package supported in R was used. Graphics were created in RStudio, Excel and GraphPad Prism.

### Statistical Analysis

Baseline characteristics are presented as medians for non-normally distributed data and percentages for categorical data. Statistical analyses were performed in GraphPad Prism or R. Graphics were created in GraphPad or R. Kaplan-Meier statistics have been used to assess the percentage of patients who were event and progression free at predetermined follow-up time points. Log-rank analysis was used to compare between disease phenotypes. Statistical significance was determined for ‘pre-AHSCT vs time point’ by Wilcoxon matched-pairs signed rank test (non-parametric, two-tailed, p < 0.05) with Bonferroni corrections for multiple tests employed where appropriate through the immunological analysis. Longitudinal time points were compared using a linear mixed-effects model to account for the possibility of missing data points including the reduction in patient numbers beyond 24 months.

## Results

### CD4+ and CD8+ T Lymphocyte Repopulation Continues to 36 Months Following AHSCT

Immune reconstitution analysis with multicolour flow cytometry was assessed in 19 relapsing-remitting MS patients for 24 months following AHSCT, with a further analysis on 13 of the cohort undertaken at 36 months. Baseline characteristics of patients and control subjects are outlined in **Supplementary Table 6**.

**Figure 1:**
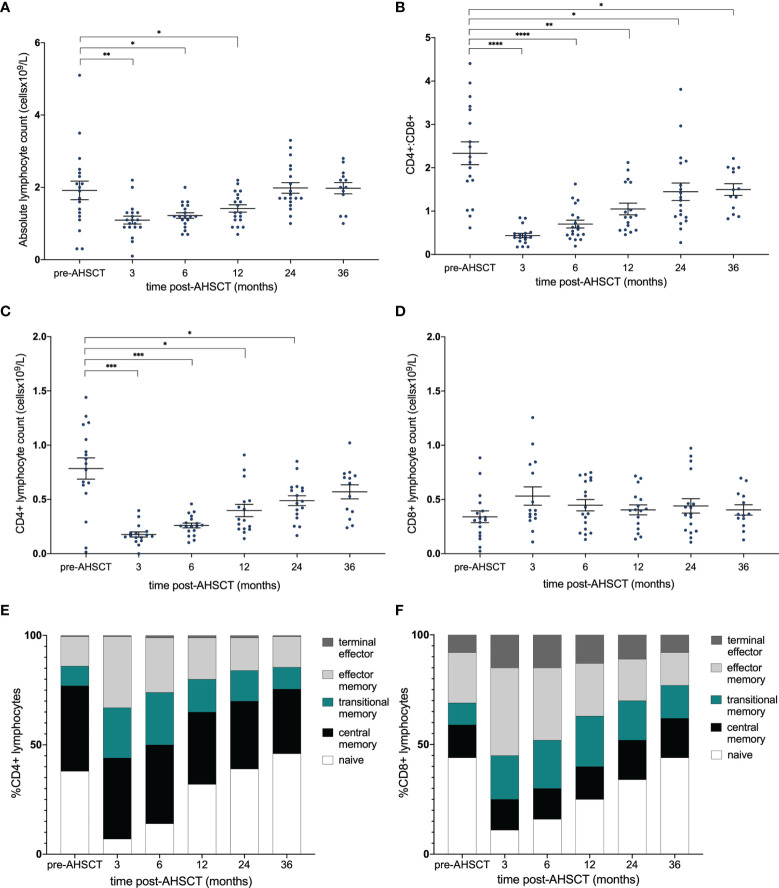
Immune reconstitution following AHSCT. **(A)** Absolute lymphocyte count (x10^9^/L) and **(B)** CD4+:CD8+ ratio pre- and post-transplant. **(C)** CD4+ and **(D)** CD8+ populations [cell count (x10^9^/L)] and stacked bar graph of the proportions of **(E)** CD4+ and **(F)** CD8+ subsets within the repertoire at each time point. **(A)** Wilcoxon signed-rank test was used to compare all time points to baseline, accounting for the reduction in patient numbers at 36 months. **(B–D)** A linear mixed model was used to detect significance (p <0.05) between each time point with the pre-transplant (control) sample. Holm-Sidak’s multiple comparison test was applied, accounting for the reduction in patient numbers at 36 months. *p < .05, **p < 0.025, ***p < 0.005, ****p < 0.0001.

Following AHSCT the absolute lymphocyte count (ALC) reached a nadir at 3 months and normalised by 24 months post-AHSCT (**Figure 1A**). However, at 36 months post-procedure the CD4+:CD8+ ratio remained significantly below baseline (p = <0.05) (**Figure 1B**) reflecting a slow recovery in the absolute number of CD4+ cells (**Figure 1C**) compared to relatively stable numbers of CD8+ lymphocytes (**Figure 1D**). Whilst repopulation kinetics appear similar between the CD4+ and CD8+ pool (**Figures 1E, F**) it is evident that an early proliferation of (effector and terminal effector) memory T-cells maintains CD8+ cell numbers (**Figure 1F**) whilst the recovery of CD4+ lymphocyte counts relates to eventual re-emergence of naïve CD4+ lymphocytes (**Figure 1E**). Stability of CD8+ cell numbers out to 36 months post-AHSCT despite an increase in the proportion of naïve cells over time implies that many memory CD8+ clones undergo attrition following naïve lymphocyte development.

### Dynamic Changes in Dominant CD4+ and CD8+ Memory Clonotypes to 36 Months Post-AHSCT

**Figure 2:**
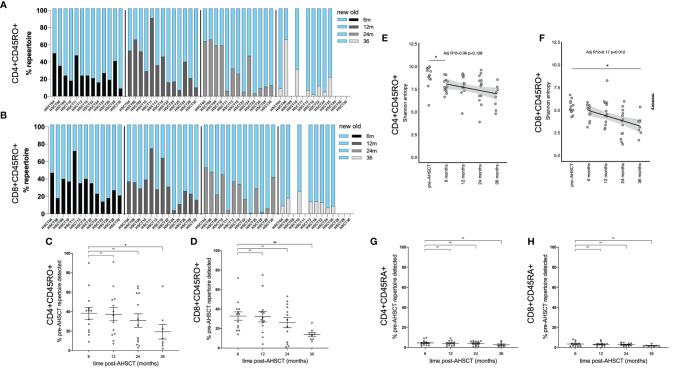
Kinetics of CD4+ and CD8+ memory clones following AHSCT. The proportion of the TCR repertoire that was new (blue) compared to baseline (pre-AHSCT) at 6, 12, 24 and 36 months and persistent (also detected pre-AHSCT) at 6 (black), 12 (dark grey), 24 (light grey) and 36 (silver) months for each individual is depicted in the CD4+CD45RO+ repertoire **(A)** and CD8+CD45RO+ repertoire **(B)**. Mean clonal persistence of the top 100 clones pre-transplant in the **(C)** CD4+CD45RO+ population and **(D)** CD8+CD45RO+ population at 6, 12, 24 and 36 months post-AHSCT. A progressive decline in the persistence of the dominant clones is observed. Diversity in **(E)** CD4+CD45RO+ and **(F)** CD8+CD45RO+ populations in the months following AHSCT. A significant downward trend, and drop between pre-AHSCT and 36 months was seen in entropy scores of CD8+CD45RO+ population post AHSCT. In the CD4+CD45RO+ population a significant drop in diversity occurs in the first 6 months, then values were not significantly different to pre-AHSCT. Mean clonal persistence of the dominant clones pre-transplant in the **(G)** CD4+CD45RA+ population and **(H)** CD8+CD45RA+ population at 6, 12, 24 and 36 months post-AHSCT. Bar/error bars in **(A, B)** show mean +/- SEM. Mann-Whitney t-tests were conducted with a Bonferroni correction applied, resulting in a significance level set at *p* < 0.025. *p < 0.025, **p < 0.005.

Having characterised lymphocyte reconstitution by flow cytometry, temporal changes in TCR clonality were investigated using high-throughput TCR sequencing. TCRβ mRNA was sequenced to 0.5 million reads on average from 10^5^ - 10^6^ sorted CD4+ and CD8+ naïve (CD45RA+) and memory (CD45RO+) cells. The dominant (top 100) CD4+ and CD8+ memory clones (by read size) were analysed longitudinally in 13 patients (n = 13 to 24 months, 8 to 36 months). A significant change in the dominant clonal landscape was evident following AHSCT (**Figures 2A, B**). An average of 40.8% (CD4+CD45RO+) and 32.8% (CD8+CD45RO+) of clones from the dominant pre-transplant repertoire were detected at 6 months, yet only 19% (CD4+; p<0.025) and 13% (CD8+; p<0.005) of the top 100 clones pre-transplant were still detected at 36 months (**Figures 2C, D**). Diversity (defined by Shannon entropy) of the CD4+CD45RO+ pool trended down following AHSCT (**Figure 2E**, adj r^2^ = 0.06, p = 0.128) but was not significantly different from baseline after 12 months and repertoire diversity of the CD8+CD45RO+ population progressively declined post-transplant (**Figure 2F** adj r^2^ = 0.17, p = 0.012); reaching a value significantly below baseline by 36 months. The proportion of the TCR repertoire that was persistent pre- and post-transplant was then assessed in the naïve compared with memory populations (**Figures 2G, H**). Based on the diversity of the naïve repertoire, prior studies looking at clonal persistence (34) and pilot studies showing minimal overlap of the naïve repertoire in biological replicates, we hypothesised only a fraction of clones would be shared over time, which was the case in both CD4+ and CD8+ naïve populations.

It has been shown that the dominant T cell repertoire is typically stable in a healthy individual over an interval period of 3 years (37, 38). To validate the changes in our AHSCT treated cohort, we assessed stability of dominant clones in MS patients on an alternate high-efficacy therapy, natalizumab. In contrast to transplant patients, an analysis of the dominant memory lymphocyte repertoire and T-cell diversity in three natalizumab treated patients (**Supplementary Table 6**) confirmed relative clonal stability over a 2-year period on treatment, with a mean of 86% (81 – 95%) of dominant CD4+CD45RO+ clones and 72% (71 – 74%) of dominant CD8+CD45RO+ clones detected in samples collected 24 months later (**Supplementary Figure S4A–C**) and no overall change in repertoire diversity over time (**Supplementary Figure S4D**). Within the naïve natalizumab populations over time very little clonal persistence was detectable (**Figure S4E**).

### Public CD4+ Clones Decrease Following AHSCT Whilst Public CD8+ Clones Increase as a Proportion of the TCR Repertoire

**Figure 3:**
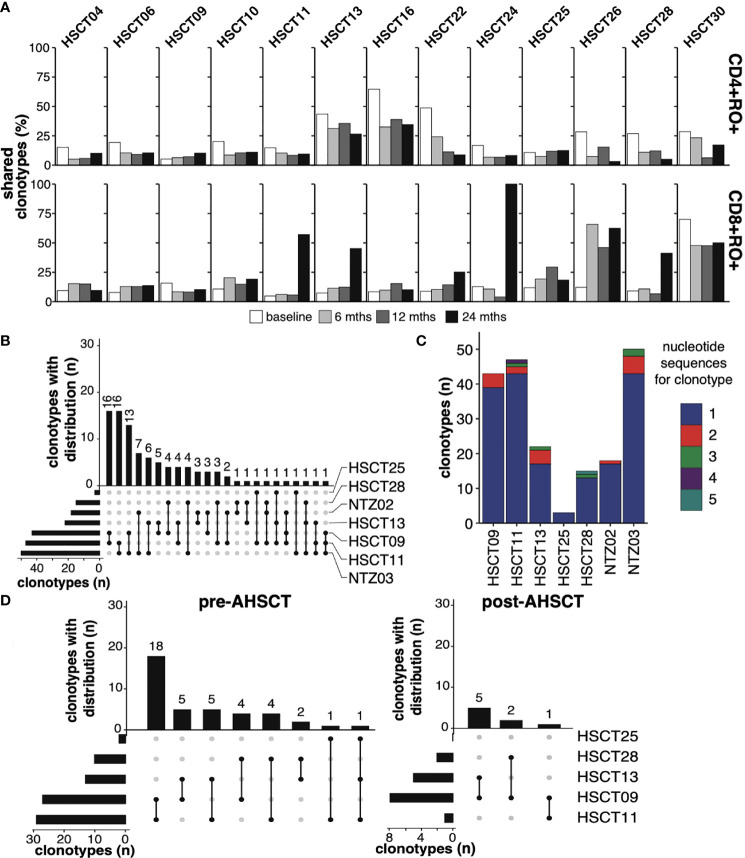
Public T-cell clones **(A)** The fraction of public T-cell clonotypes within CD4+CD45RO+ [top panel] and CD8+CD45RO+ [bottom panel] of each individual (top x-axis, each horizontal subsection) undergoing AHSCT at baseline (0, white), 6 (light grey), 12 (dark grey) and 24 months (black, bottom x-axis). **(B)** Upset plot depicting patterns of sharing of public CD4+CD45RO+ clones (minimum 2 reads in each individual) amongst the 7 HLA DRB1*15:01 positive patients at baseline, ranked by the number of clonotypes shared between *1501-positive individuals. The vertical bars, ‘clonotypes with distribution’, represent the number of clones (not clone size) shared between the individuals linked in the dots below each bar. The horizontal bars to the lower left depict the number of public clones within each individual’s repertoire, ranked from least number of public clones to most. **(C)** The number of public clones within each individual depicted by different nucleotide reads, 1 to 5 (maximum) e.g. within HSCT09, 39 public clones were identified, each encoded by a different nucleotide sequence, whilst 4 clones were identified that were each encoded by two different nucleotide sequences. 12% of public clones in (6 of the 7) *15:01-positive individuals are encoded by differing nucleotide sequences. **(D)** Upset plots depicting patterns of sharing of public CD4+CD45RO+ clones (minimum 2 reads in each individual) amongst 5 HLA DRB1*15:01-positive patients undergoing AHSCT at baseline and at 24 months, ranked by the number of clonotypes shared between individuals. No shared clones were identified in HSCT25 post-AHSCT.

Despite converging evidence of a ‘public’ T lymphocyte population in the pathogenicity of MS the kinetics of public T cell clones in response to immunotherapy is poorly characterised. Samples from pre-, 6-, 12- and 24-months post-AHSCT in 13 patients were analysed for the presence of clone sharing. Attrition of the CD4+ memory population at 24 months was mirrored by a decline in the proportion of public CD4+ memory clones (**Figure 3A**). At baseline, a total of 181 public CD4+ memory clones were identified in patients undergoing AHSCT. This declined to 89 clones at 24 months. Despite restricted diversity in the CD8+ memory repertoire at 24 months there was a mean increase in the fraction of the TCR repertoire which was public following AHSCT (**Figure 3A**). The number of public clones in the CD8+ memory population increased from 48 clones pre-AHSCT to 59 at 24 months post-AHSCT. Although some individuals appeared to share a greater proportion of their TCR repertoire at all timepoints (**Figure 3A**), no common clinical characteristics could be identified across those patients. The T cell repertoire was annotated for reported antigen specificity, with restrictions of V gene, J gene and CDR3 amino acid sequence where the recorded HLA allele matched the donor MHC haplotype. Analysis of all sample time points established that between 0.21 – 3.11% of the TCR repertoire could be annotated for viruses with the majority of identifiable virus-specific clones reactive to EBV or CMV epitopes (**Supplementary Figure 5**). Generally, the proportion of virus specific clones remained relatively stable in the CD4+ repertoire over time, whilst the CD8+ repertoire demonstrated an early increase in the proportion of T cells with viral reactivity, which spiked again at 24 months (**Supplementary Figure 5**). Similar trends were observed in the public TCR profiles of patients, and the increase in the proportion of public CD8+ memory clones in certain individuals (**Figure 3A**) could not be readily explained by TCRs with reported affinity for common viruses (**Supplementary Figure 6**). No definable patterns were seen in clones annotated for self-antigen from the VDJdb. No centralised repository for TCR sequences identified in MS patients exists, however our data was screened against a publicly available dataset from a similar study (25). 13.7% of public clones in our data set could be identified in the accessed data.

Next the TCR repertoire was analysed using the GLIPH2 algorithm (55), with the aim of identifying amino acid motifs which may signify a TCR ‘signature’ of MS. Pre-AHSCT TCRs (including clones shared with baseline samples from the natalizumab-treated cohort) were compared with a reference healthy population (58, 59), and then compared with post-transplant timepoints. Whilst some enriched TCR motifs were detected in the pre-transplant MS population compared with healthy controls, these were mostly restricted to individual patient samples, and no unifying TCR motif was significantly enriched within either the CD4+ or CD8+ memory TCR repertoire of the whole cohort (data not shown).

Public CD4+CD45RO+ T cell clones have never been probed in an HLA DRB1*15:01 positive MS cohort. Seven patients within the MS cohort (AHSCT = 5/13, NTZ = 2/3) were identified to express the class II MS-risk allele DRB1*15:01, and clone sharing in this population was therefore explored further. The complete HLA type of all patients is outlined in **Supplementary Table 7**. Ninety public CD4+CD45RO+ clones were detected in HLA DRB1*15:01-positive patients, with maximum sharing across three individuals (**Figure 3B**). Again, no significantly enriched CDR3 motif was identified within the CD4+CD45RO+ pool across HLA DRB1*15:01-associated public clones compared with the reference population. Consistent with the hypothesis of public clone generation relating to convergent recombination (60), twenty-three of these public clones were encoded by more than one nucleotide sequence, with a maximum of 5 nucleotide sequences encoding a public TCR clone (**Figure 3C**). Eleven of these clones could be detected in ‘non-*15:01’ individuals at baseline. Forty public CD4+CD45RO+ clonotypes were detected within the five *15:01-positive patients undergoing AHSCT, with only 8 public clones detected at 24 months (**Figure 3D**). Two of the 8 clonotypes were present in the public CD4+CD45RO+ population pre-AHSCT and 6 were new. The two persisting public clones did not correlate with recurrent disease activity.

### AHSCT Depletes Public CD4+ Memory Clonotypes Including Those With Potential Autoreactivity

An analysis was then performed to assess TCR production pre- and post-AHSCT in regard to the ‘probability of generation’ and thymic selection pressure on T lymphocytes. Thymic generation of TCRs through V-D-J recombination, nucleotide additions and/or substitutions involves a degree of ‘non-randomness’ (60). TCRs that are more readily generated are mostly germline-encoded (fewer nucleotide insertions and deletions), whilst those that are less frequently generated by the recombination process include more extensive junctional processing. Stochastically, more-readily generated clones are more likely to be public. CD4+ and CD8+ clones were subjected to an analysis utilising a novel modelling tool entitled SONIA to obtain values for P_GEN_ (probability that the sequence is generated by V-D-J recombination) and P_POST_ (probability of the clonotype surviving thymic selection); with lower values indicating a ‘rare’ clone (39, 56, 57).

**Figure 4:**
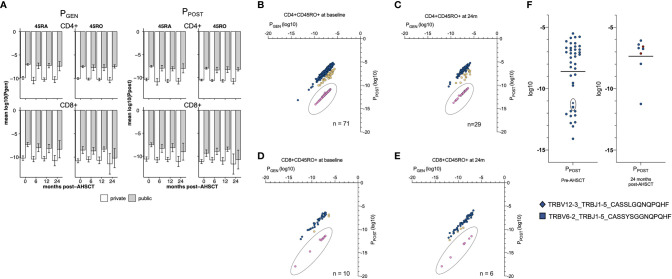
Probability values T cell profiles. **(A)** The average P_GEN_ (left panel) and P_POST_ (right panel) of public and private clonotypes. Y-axis is mean log10. For CD4+ and CD8+ naïve and memory populations. CD4+ (left hand side) and CD8+ (right hand side) CD45RA+ (top half) and CD45RO+ (bottom half) lymphocyte pools are depicted. Within each lymphocyte pool, the mean P_GEN_ and P_POST_ (bar and single standard deviation, error bar) are depicted for public (grey) and private (white) clonotypes. At all timepoints the mean P_GEN_ and P_POST_ values are greater in the public clonotypes than private clonotypes, and significantly different in all lymphocyte subsets at all timepoints (p = <0.01) excluding the CD8+ memory pool at 24 months (p = 0.062). P_GEN_ and P_POST_ values of CD4+CD45RO+ **(B)** and CD8+CD45RO+ **(C)** public clonotypes at baseline and 24 months post-AHSCT **(D, E)**. The P_GEN_ : P_POST_ ratio was calculated for each clone. The P_GEN_ : P_POST_ ratio ranged from 1:1.38 to 1:7.4x10^-7^. Clones were then binned; 1:1.38 – 0.1 (blue, not rare), 1:0.1 –1 x 10^-4^ (yellow, intermediate) and 1:<1x10^-4^ (pink, rare to survive thymic negative selection). Clones with a P_GEN_ : P_POST_ ratio 1: <1x10^-4^ (pink) were considered a population of interest for possible self-reactive T cells. All public clones with a P_GEN_ : P_POST_ ratio 1: <1x10^-4^ had a P_POST_ value of <-10log10. **(F)** P_POST_ values of public CD4+CD45RO+ clones in HLA DRB1*15:01 positive individuals pre- and 24 months post AHSCT. Within the 24-month plot, clones that were detected in the public repertoire at baseline have been depicted in dark red, n = 2. Two clones with a low P_POST_ value (square, diamond) and significant sequence homology - sharing the GXNQPQHF motif – identified pre-AHSCT were undetectable at any time point in any patient post-AHSCT and were also detected in NTZ02. Error bars (black) depict the mean. Using a Mann-Whitney unpaired non-parametric t-test there was no significant change between mean probability values pre- to post-AHSCT.

At all study timepoints, public TCRs had a higher mean P_GEN_ than an individual’s corresponding private clonal profile, confirming that public TCRs are more ‘likely to be generated’ by any individual (**Figure 4A**). Similar average P_GEN_ and P_POST_ values were observed in CD4+ and CD8+ populations pre- and post-AHSCT despite the profound changes in the dominant TCR repertoires that were observed over the study period (**Figure 2**). The P_GEN_ and P_POST_ values were then utilised to determine the likelihood of detection of clones in the peripheral circulation. Clonotypes with a high P_GEN_ to P_POST_ ratio (**Figures 4B–E**, pink) were considered as possible escapees of negative thymic selection given they are ‘easily generated’ in the thymus yet less likely to reach the circulation. This population typically had P_POST_ values <-10log_10_ and were considered a population of interest for further exploration of putative pathogenic public clones.

Utilising these metrics, we performed an exploratory analysis of public clonotypes which may be relevant to MS pathogenesis, focusing on public CD4+CD45RO+ clones within HLA DRB1*15:01 positive patients as we hypothesised this would provide the greatest likelihood of defining pathogenic clones. Public clonotypes were therefore analysed in the context of clinical response. Of the forty public clonotypes present in this cohort pre-AHSCT, eleven had a P_POST_ value of <-10log_10_ (**Figure 4F**). Twenty-four months post-AHSCT, of the 8 public CD4+CD45RO+ clonotypes in *15:01 positive individuals, only one had a P_POST_ value <-10log_10_. Two clones detected pre-AHSCT and not at 24 months (CASSLGQNQPQHF and CASSYSGGNQPQHF) demonstrate significant sequence homology with a shared GXNQP(QHF) motif and both clones were also identified in NTZ02 at baseline.

### T-Cell Receptor Motifs Analysis Identifies CD4+CD45RO+ Clones That Proliferate in the Lymphopenic Environment Post-AHSCT

**Figure 5:**
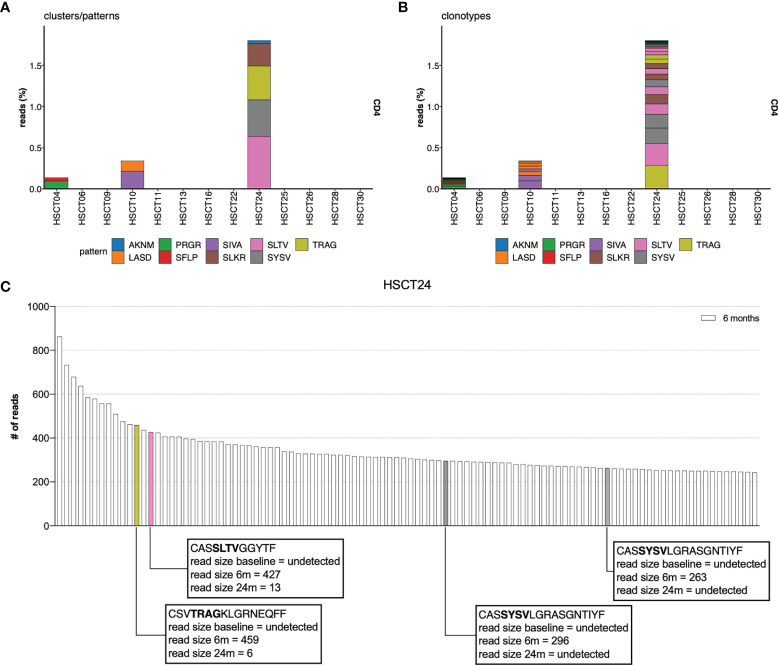
TCR CDR3 motif analysis. TCR CDR3 motif analysis compares the 6-month CD4+ TCR repertoire to the 0 (pre), 12, 24 and 36 months for AHSCT subjects. Significantly enriched motifs were identified only in the CD4+CD45RO+ population at 6 months in relation to pre-AHSCT. **(A)** The proportion of overall read count that included each enriched motif cluster. **(B)** The proportion of clonotypes within each individual at 6 months which contain enriched motifs. **(C)** Identifying specific T-cell clones undergoing LIP in patient example HSCT24. Enriched motifs were detected in 4 of the top 100 clones at 6 months. Clones containing enriched motifs at 6 months have been colour coded to their relevant motif eg: SLTV coded pink. CASSYSVLGRASGNTIYF is encoded by two different nucleotide sequences. Read size pre- and 24-months post-AHSCT has been annotated.

Approximately half of the CD4+ and CD8+ memory clones that dominate the TCR repertoire 6 months following AHSCT were not present in the dominant pre-transplant memory repertoire. We therefore sought to determine whether certain TCRs would have a binding avidity that would select for proliferation under lymphopenic conditions, and whether these were low frequency memory clones or naïve clones. An analysis employing the GLIPH2 algorithm (55), comparing TCR motifs within CD4+ and CD8+ memory repertoires at 6 months with corresponding pre-transplant, 12- and 24-month post-transplant repertoires identified significantly enriched motifs in the CD4+CD45RO+ populations of 3 individuals at 6 months compared to baseline (**Figure 5A**). These motifs were identified and enriched in multiple clones within the individual’s TCR repertoire (**Figure 5B**). All 3 patients were deemed ‘responders’ with no clinical or radiological disease activity post-AHSCT. Two of the three patients had improved EDSS scores following AHSCT. Most clones with enriched motifs could not be detected in the pre-AHSCT CD4+ memory repertoire, suggesting they were derived from a naïve population (**Figure 5C**). Despite evidence of lymphopenic induced proliferation of CD8+ memory cells (**Figure 1F**) and prior work suggesting this is the dominant homeostatic mechanism for recovery from lymphopenia, no significantly enriched motifs were identifiable in the CD8+CD45RO+ population at 6 months compared to baseline.

### Recovery of the Thymically-Derived Naïve T-Cell Repertoire Assists Diversity Post-AHSCT

**Figure 6:**
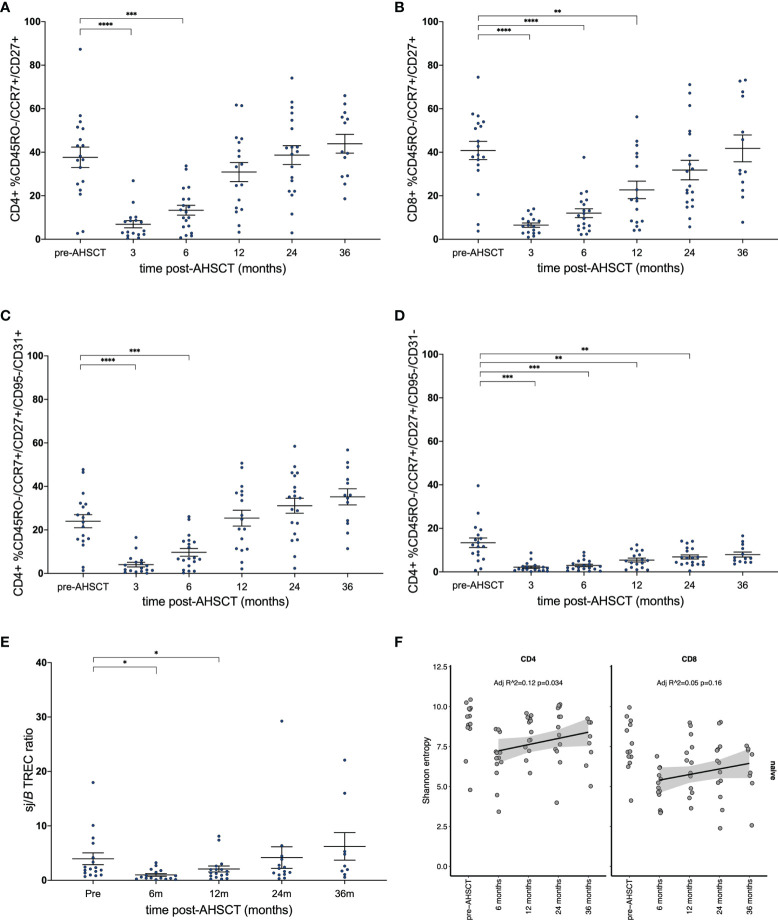
Reconstitution of naïve T-cell subsets. **(A)** CD4+ and **(B)** CD8+ naïve T-cell populations prior to and following AHSCT. **(C)** CD4+ T lymphocytes with signatures of recent thymic egress (CD31+) drive the recovery of CD4+ naïve populations, whilst **(D)** non-RTE naïve T-cells remain below baseline up to the 36-month time point. **(E)** Longitudinal sj/βTREC ratios in patients pre- and post-AHSCT. **(F)** Clonal diversity as measured by Shannon entropy in the CD4+CD45RA+ population (left) and CD8+CD45RA+ (right). **(A–E)** Bar/error bar denotes median and inter-quartile range. **(A–D, F)** A linear model was used to detect significance (p <0.05) between each time point with the pre-transplant (control) sample. **(E)** Wilcoxon signed-rank test was used to compare all time points to baseline. Holm-Sidak’s multiple comparison test applied. *p < .05, **p < 0.025, ***p < 0.005, ****p < 0.0001.

**Figure 7:**
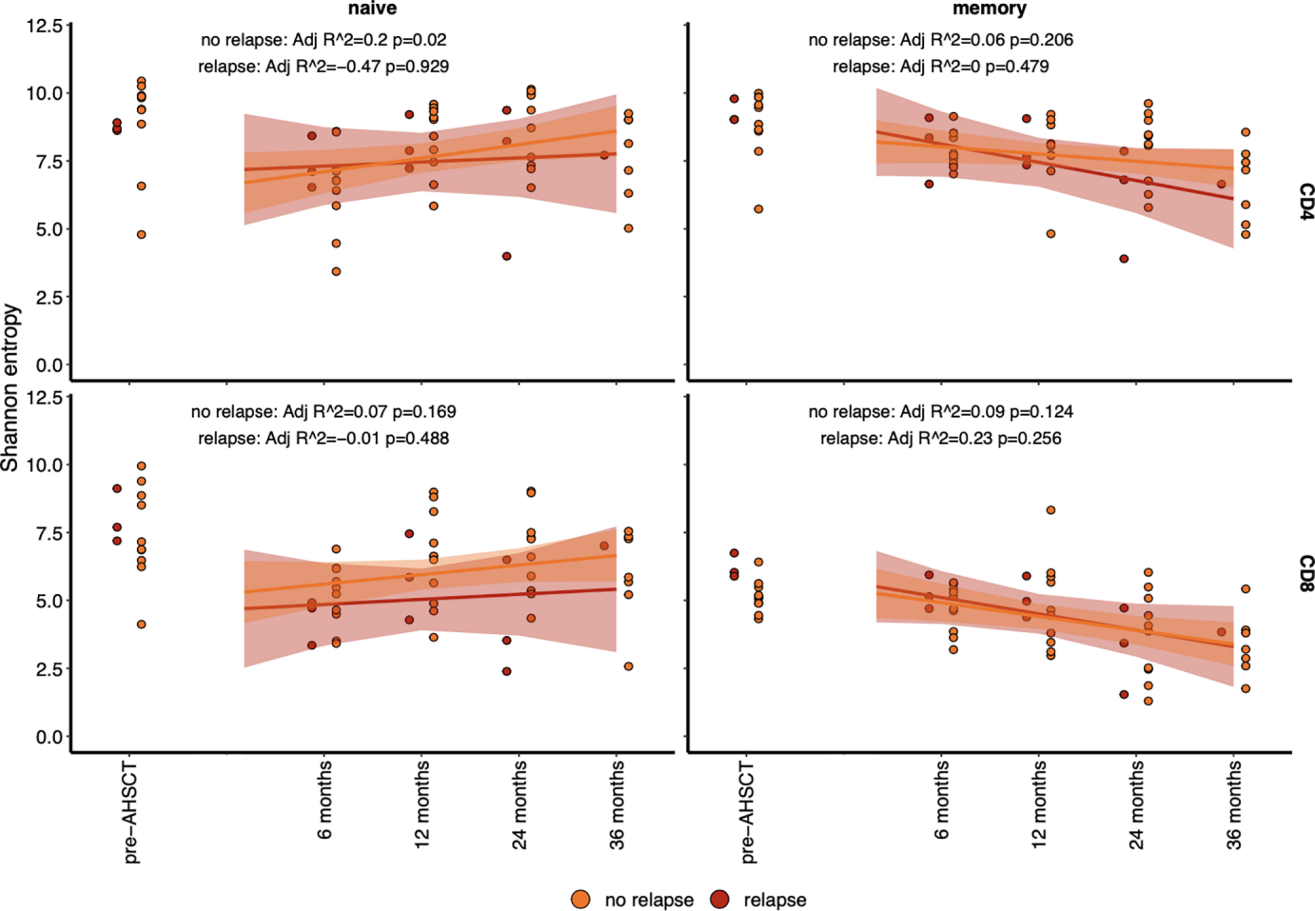
Diversity (as determined by Shannon entropy) in lymphocyte subsets analysed by relapsed (red) patients and non-relapsed (orange) patients. The entropy of naïve (CD45RA+) population (left) and memory (CD45RO+) population (right), with CD4+ (top) and CD8+ (bottom) have been plotted before and after AHSCT. A line of fit, and significant for each population is depicted per panel. The only significant line of fit, with an increase in diversity is in the CD4+CD45RA+ population.

Flow cytometry analysis of the CD4+ naïve T cell population confirmed a return to pre-transplant proportions by twelve months post-AHSCT (**Figure 6A**), while the corresponding CD8+ population did not recover until 24 - 36 months (**Figure 6B**). The recovery of the CD4+ naïve population was driven by a surge in the CD31+ recent thymic emigrant (RTE) population (**Figure 6C**), whilst the CD4+CD31- naïve population (**Figure 6D**) remained below baseline at all time points following AHSCT until 36 months. Unfortunately, no reliable method exists for the quantification of thymic derived CD8+ lymphocytes (61, 62). Overall thymic recovery was not significantly impacted by age, sex, disease duration or mean dose of infused CD34+ cells. Extended thymopoiesis was confirmed by TREC quantification (**Figure 6E**), where mean sj/β TREC values demonstrated recovery by 24 and a trend to exceeding baseline by 36 months (baseline mean ratio 3.96; 4.18 at 24 months p = 0.84, 6.24 at 36 months p = 0.38). Shannon entropy was observed to increase in the CD4+ naive population following the 6-month nadir (Adj r ^2^ = 0.12, p = 0.034) but was not significantly different by 12, 24 or 36 months compared with pre-transplant (**Figure 6F**) highlighting the duration required post-transplant to re-establish a varied naïve T cell pool. In the CD8+ naïve population, a non-significant recovery of diversity was evident out to 36 months. Lastly, we sought to define whether recovery of diversity varied between patients who relapsed (new clinical and/or MRI activity) and responders (**Figure 7**). When divided into these two subgroups the diversity trends between 6 to 36 months lost significance in all but one lymphocyte subset. A progressive increase in entropy was evident in the CD4+CD45RA+ repertoire from the 6 month nadir in responder patients, without a significant upward trend in non-responders.

## Discussion

AHSCT is administered in MS as a highly efficacious immune reconstitution treatment in autoimmune disease (63, 64), with therapeutic effects extending beyond the period of lymphopenia (65). An understanding of the immunology that underpins AHSCT in MS is essential to the development of future immunotherapies that may supersede ablative chemotherapy. In this study, patterns of immune reconstruction were consistent with other studies of AHSCT in MS (24, 26–28). However, the inclusion of a 36-month time point in this study establishes that a reduced CD4+:CD8+ ratio extends beyond the recovery of absolute lymphocyte counts, suggesting that restoration of immune homeostasis extends beyond three years following AHSCT.

Muraro et al. (25) suggested that lymphopenia in the first 12 months post-AHSCT reflected depletion of CD4+ lymphocytes, and that early lymphocyte recovery was due to lymphopenic induced proliferation of CD8+ cells. In this study, with sorted lymphocyte subsets, we confirm an early expansion of CD8+ memory populations, with continual decline in diversity of this population post-AHSCT, hinting that memory lymphocytes undergo clonal attrition following lymphopenic induced proliferation (LIP). In contrast to prior publications, we demonstrate that LIP also occurs, albeit to a lesser degree, in CD4+ memory populations with a mirrored effect on diversity and a significant change in the dominant memory repertoire at 36 months. Interestingly, motif analysis did not detect significantly enriched CD8+CD45RO+ TCR motifs in the early post-transplant period (6 months) compared to baseline, possibly because early lymphopenic induced proliferation of CD8+ cells arises from clones that were already dominant pre-AHSCT. Conversely in the CD4+CD45RO+ pool significantly enriched TCR motif representation was detected in three patients at 6 months post-transplant.

Whilst foreign antigen-driven lymphocyte expansion cannot be excluded in these three patients, we favour the interpretation that the lymphopenic environment, with increased levels of IL-7 and IL-15 (66, 67), promotes homeostatic expansion and acquisition of the CD45RO+ memory phenotype in TCRs of specific avidities to self-antigen. This hypothesis of classical lymphopenic induced proliferation is supported by the observations that the majority of the CD4+45RO+ clones with enriched TCR motifs arise *de novo* post-AHSCT suggesting a conversion from naïve to memory phenotype. Enriched motifs were identified in three responder patients. While this study did not investigate T regulatory cells it is notable that multiple studies have detected a transient surge in CD4+ T regulatory cells in the first year following AHSCT (26, 28, 43) and other lymphoablative MS therapies (68–70), including a diversification of the Treg repertoire (71). Given inducible Tregs are defined by avidity to self-antigen (72), it is possible the lymphopenic environment post-AHSCT directly contributes to the regeneration of immune tolerance by favouring their proliferation.

To date, only two studies have analysed the kinetics of clonal lymphocytes following AHSCT for MS, with neither study tracking populations longitudinally, nor probing the repertoire profiles for public clones. Analysis at 6, 12, 24 and 36 months in this study demonstrated dynamic changes in the dominant CD4+CD45RO+ and CD8+CD45RO+ clonal repertoire. It is shown here that despite the ‘single shot’ approach of AHSCT, TCR clones continue to be depleted over the post-transplant period up to 36 months. By 36 months there has been a major shift in the CD4+ memory TCR repertoire including depletion of many public clones. Conversely there is an increase in the number and proportion of CD8+ public clones at 24 months, although we were unable to define the affinity of many of these clones.

It is evident that generation and selection of TCRs within the thymus influences an individual’s T-cell repertoire by stochastic means. Algorithms such as SONIA provide a method to quantify the variability of the ‘probability of generation’ and ‘probability of survival to the periphery’ within an individual’s TCR profile, previously untenable due to sampling limitations. Despite public clones in general having higher P_GEN_ and P_POST_ values than individual clones, an exploratory analysis helped characterise public clonotypes with possible characteristics of escaping thymic negative selection, i.e. a low P_GEN_ to P_POST_ ratio and P_POST_ values <-10log_10_ for a pilot analysis of pathogenic clones. Here, within the CD4+CD45RO+ pool of *15:01 positive individuals, we identified two clonotypes with sequence homology (a shared GXNQPQHF motif) which were undetectable following AHSCT. Acknowledging the exploratory nature of this analysis, we suggest that this methodology may prove useful in future immune repertoire studies of autoimmune diseases.

The re-development of a diversified naïve T-cell repertoire lacking auto-reactive T-cells yet capable of mounting an immune response to antigenic challenge is key to the reconstitution of a functional immune system post-AHSCT. Only a fraction of the naïve TCR repertoire persisted between baseline and 24 months post-AHSCT, yet this finding was felt to relate not only to clonal deletion and thymic repopulation but inherent limitations on sampling the breadth of the naïve repertoire (73). Following transplant, circulating numbers of thymically derived naïve CD4+ naive lymphocytes normalise by 24 months, but TCR diversity of this population remains below pre-transplant levels at 36 months, apparently independent of patient age. Importantly, relapsed patients did not demonstrate a significant recovery of naïve TCR repertoire diversity. Despite extending the follow-up duration from previous TCR sequencing studies in this field, a limitation of this study is the smaller number of patients followed to 36 months. Longer duration studies are required to assess whether the naïve population becomes more diverse with time, or whether diversification of specific lymphocyte phenotypes relates to clinical outcome (71). Additionally, the efficacy of AHSCT in controlling relapses and new MRI activity has limited our ability to correlate patterns of immune reconstitution with disease activity. Lastly, we were unable to identify clear correlates between immunological parameters and improvement in disability scores. Whilst the post-AHSCT environment is opportune to probe for the re-emergence of clones that associate with relapsing disease, future work will require collaborative analyses to better address this issue.

This study concludes that T-cells remain in a state of dynamic flux three years post-AHSCT in MS patients. Early expansion of dominant CD8+ memory lymphocytes and a conversion from a naïve to memory phenotype of CD4+ lymphocytes counters cytotoxic lymphodepletion. By 36 months post-transplant the dominant CD4+ and CD8+ memory TCR repertoire is significantly different from pre-transplant. Restricted diversity of both memory populations is evident, possibly due to chemotherapy induced ablation and subsequent post-proliferative clonal attrition. Gradual recovery of diversity in the naïve population relates to thymic output, out to 36 months post-transplant. Consistent with reshaping of the memory lymphocyte repertoire is a reduction in the number of public CD4+ clones at 24 months post-AHSCT. Finally, we outline a novel methodology for analysing TCR repertoires for clones characteristic of autoimmunity. This work demonstrates that AHSCT induces sustained periods of disease remission through dynamic changes in clonal T cell repertoire out to 36 months post-transplant – suggesting an ongoing process that may be responsible for the profound and sustained remissions provided by this therapy.

## Data Availability Statement

The datasets presented in this study can be found at NCBI’s SRA. Data is deposited under BioProject: PRJNA721253 and can be accessed at https://www.ncbi.nlm.nih.gov/bioproject/PRJNA721253

## Ethics Statement

The studies involving human participants were reviewed and approved by St Vincent’s Hospital Sydney HREC. The patients/participants provided their written informed consent to participate in this study.

## Author Contributions

Conceptualisation: JMa, MS, FL, DM, JMo, and IS. Methodology: JMa, KJ, MS, CF, MK, JZ, RC, and FL. Investigation: JMa, KJ, MS, BH, CF, MK, KH, and BCD. Visualisation: JMa, KJ, and IS. Funding acquisition: JMa, DM, and JMo. Project administration: BW, DM, JMo, and IS. Supervision: DM, JMo, and IS. Writing – original draft: JMa and IS. Writing – review and editing: JMa, KJ, MS, BW, MK, KH, JZ, RC, DM, JMo, and IS. All authors contributed to the article and approved the submitted version.

## Funding

MS Research Australia Postdoctoral Fellowship 19-0690 (JM).

## Conflict of Interest

JMa and IS have received honoraria from Biogen, Roche, Sanofi Genzyme, Merck and Teva. Subsequent to involvement in the research, CF is now employed at BD Biosciences.

The remaining authors declare that the research was conducted in the absence of any commercial or financial relationships that could be construed as a potential conflict of interest.

## Publisher’s Note

All claims expressed in this article are solely those of the authors and do not necessarily represent those of their affiliated organizations, or those of the publisher, the editors and the reviewers. Any product that may be evaluated in this article, or claim that may be made by its manufacturer, is not guaranteed or endorsed by the publisher.
