# Supplementary material for: Haematopoietic Stem Cell Transplantation Results in Extensive Remodelling of the Clonal T Cell Repertoire in Multiple Sclerosis
Source: Front Immunol. 2022 Feb 7;13:798300. doi: 10.3389/fimmu.2022.798300 (PMC8859174; doi:10.3389/fimmu.2022.798300)
Supplement: Supplementary file 1 [file DataSheet_1.docx]

**Supplementary material:**

Supplementary Table 1 (Methods)

Supplementary Table 2 (Methods)

Supplementary Table 3 (Methods)

Supplementary Table 4 (Methods)

Supplementary Table 5 (Methods)

Supplementary Table 6

Supplementary Table 7

Supplementary Figure 1 (Methods)

Supplementary Figure 2 (Methods)

Supplementary Figure 3 (Methods)

Supplementary Figure 4

Supplementary Figure 5

Supplementary Figure 6

Supplementary - Methods Table 1: Key Resources

| **Antibodies and beads** |  |  |
| --- | --- | --- |
| Fluorochrome/Antibody |  |  |
| - AF700/FVS700 | BD Biosciences | Cat#564997 |
| - APC-H7/CD8 | BD Biosciences | Cat#560179 |
| - APC/CD4 | BD Biosciences | Cat#340443 |
| - BB515/CD95 | BD Biosciences | Cat#564596 |
| - BV421/CD27 | BD Biosciences | Cat#562513 |
| - BV480/CD31 | BD Biosciences | Cat#566144 |
| - BV650/CD45RO | BD Biosciences | Cat#563750 |
| - BV786/CD45RA | BD Biosciences | Cat#563870 |
| - PE-CF594/CCR7 | BD Biosciences | Cat#562381 |
| - PE-Cy7/CD3 | BD Biosciences | Cat#557851 |
| Brilliant stain buffer | BD Biosciences | Cat#563794 |
| CompBeads - Anti-Mouse Ig/Negative control set | BD Biosciences | Cat#552843 |
| CS&T Beads | BD Biosciences | Cat#656505 |

Supplementary - Methods Table 2: PBMC staining for T cell reconstitution panel.

| **Laser** | **Antibody** | **Fluorochrome** | **Volume µl/test** | **Volume – isotype µl/test** | **Incubation duration (mins)** |
| --- | --- | --- | --- | --- | --- |
| R730/45 | FVS700 | AF700 | - | - | - |
| YG610/20 | CCR7 | PE-CF594 | 5 | 5 | 15 @ 37°C then  15 @ room temperature (RT) |
| V450/50 | CD27 | BV421 | 5 | 5 | 15 @ RT |
| V525/50 | CD31 | BV480 | 5 | 5 | 15 @ RT |
| V670/30 | CD45RO | BV650 | 5 | 5 | 15 @ RT |
| V780/60 | CD45RA | BV786 | 2.5 | 2.5 | 15 @ RT |
| B525/50 | CD95 | BB515 | 5 | 5 | 15 @ RT |
| YG780/60 | CD3 | PE-Cy7 | 3 | 3 | 15 @ RT |
| R780/60 | CD8 | APC-H7 | 0.625 | 0.625 | 15 @ RT |
| R670/30 | CD4 | APC | 0.625 | 0.625 | 15 @ RT |
| Staining buffer | - | - | 50 | 50 | - |

Supplementary - Methods Table 3: Cell surface markers for T lymphocyte populations.

| **T-cell populations of interest** |  |  |  |  |  |  |
| --- | --- | --- | --- | --- | --- | --- |
| NB: all populations of interest were analysed in both CD3+CD4+ and CD3+CD8+ populations, apart from the 'RTE' and 'non-RTE' subsets which is only recognised in the CD3+CD4+ populations | | | | | | |
| *Title* | *Abbreviation* | *Surface markers* | | | | |
| Naïve | Naïve | CD45RO- | CCR7+ | CD27+ | CD95- |  |
| RTE | RTE | CD45RO- | CCR7+ | CD27+ | CD95- | CD31+ |
| Non-RTE naïve cells | Non-RTE naïve | CD45RO- | CCR7+ | CD27+ | CD95- | CD31- |
| Central memory | CM | CD45RO+ | CCR7+ |  |  |  |
| Transitional memory | TM | CD45RO+ | CCR7- | CD27+ |  |  |
| Effector memory | EM | CD45RO+ | CCR7- | CD27- |  |  |
| Terminal effector (CD45RA+ revertant) | EMRA | CD45RO- | CCR7- | CD27- |  |  |

Supplementary - Methods Table 4: Cell surface markers for FACS.

| **FACS populations of interest** |  |  |
| --- | --- | --- |
| *Title* | *Surface markers* |  |
| CD4+ Naïve | CD4+ | CD45RA+ |
| CD8+ Naive | CD8+ | CD45RA+ |
| CD4+ Memory | CD4+ | CD45RO+ |
| CD8+ Memory | CD8+ | CD45RO+ |

Supplementary - Methods Table 5: PBMC staining for FACS panel.

| **Laser** | **Antibody** | **Fluorochrome** | **Volume µl/test** | **Incubation time (mins)** |
| --- | --- | --- | --- | --- |
| R730/45 | FVS700 | AF700 | - | - |
| V670/30 | CD45RO | BV650 | 5 | 15 @ RT |
| V780/60 | CD45RA | BV786 | 5 | 15 @ RT |
| YG780/60 | CD3 | PE-Cy7 | 3 | 15 @ RT |
| R780/60 | CD8 | APC-H7 | 1 | 15 @ RT |
| R670/30 | CD4 | APC | 2 | 15 @ RT |
| Staining buffer | - | - | 50 | - |

Supplementary Table 6. Patient cohort for analysis including immunological investigations and clinical characteristics. NTZ = natalizumab, Tx = transplant.

| **Patient ID** | **Immuno-phenotyping** | **T cell receptor sequencing** | **Sex** | **Age at Tx (or baseline)** | **HLA DRB1*15:01** | **Disease duration pre-AHSCT (months)** | **Clinical relapses in the**  **24 months**  **pre-AHSCT** | **MRIs with disease activity in the**  **24 months**  **pre-AHSCT** | **CD34+ dose (x10^6^/kg)** | **Clinical relapse or MRI activity post- AHSCT** | **EDSS pre-AHSCT** | **EDSS on last follow up (months)** |
| --- | --- | --- | --- | --- | --- | --- | --- | --- | --- | --- | --- | --- |
| HSCT02 | Yes  0/3/6/12/24/36 months | Insufficient sample | M | 27 | N/A | 130 | 2 | 2 | 9.40 | no | 4.5 | 6  (36) |
| HSCT04 | Yes  0/3/6/12/24/36 months | Yes  0/6/12/24/36 months | F | 38 | No | 127 | 3 | 3 | 4.99 | no | 4 | 1  (36) |
| HSCT05 | Yes  0/3/6/12/24/36 months | Insufficient sample | F | 52 | N/A | 188 | 0 | 2 | 6.71 | no | 6 | 6  (36) |
| HSCT06 | Yes  0/3/6/12/24/36 months | Yes  0/6/12/24/36 months | F | 35 | N | 47 | 2 | 2 | 11.75 | no | 3 | 0  (36) |
| HSCT08 | Yes  0/3/6/12/24/36 months | Insufficient sample | F | 46 | N/A | 60 | 3 | 2 | 9.54 | no | 4.5 | 4.5  (36) |
| HSCT09 | Yes  0/3/6/12/24 months  *lost to follow up at 30 months | Yes  0/6/12/24 months | F | 29 | Y | 112 | 1 | 2 | 9.84 | no | 3.5 | 3.5  (36) |
| HSCT10 | Yes  0/3/6/12/24/36 months | Yes  0/6/12/24/36 months | F | 37 | N | 179 | 1 | 1 | 7.41 | no | 4 | 1  (36) |
| HSCT11 | Yes  0/3/6/12/24/36 months | Yes  0/6/12/24 months  *primer error 36 month sample | F | 31 | Y | 62 | 1 | 0 | 5.90 | Single new non-enhancing T2/FLAIR hyperintensity on MRI at 36 months post-AHSCT. No clinical correlate. Didn’t restart DMT. | 4 | 4  (36) |
| HSCT13 | Yes  0/3/6/12/24/36 months | Yes  0/6/12/24/36 months | F | 22 | Y | 62 | 3 | 3 | 6.48 | Relapse with right leg weakness 13 months post-AHSCT. Didn’t restart DMT. | 3.5 | 2  (36) |
| HSCT14 | Yes  0/3/6/12/24 months  *censored 28 months | Insufficient sample | F | 37 | N/A | 141 | 0 | 2 | 8.73 | Relapse with L’hermittes phenomenon and lower limb weakness 14 months post-AHSCT. Restarted DMT at 28 months. | 6 | 6  (24) |
| HSCT15 | Yes  0/3/6/12/24/36 months | Insufficient sample | M | 30 | N/A | 39 | 2 | 1 | 6.41 | no | 4 | 4  (36) |
| HSCT16 | Yes  0/3/6/12/24/36 months | Yes  0/6/12/24/36 months | M | 25 | N | 8 | 4 | 3 | 7.44 | no | 2 | 2  (36) |
| HSCT22 | Yes  0/3/6/12/24/36 months | Yes  0/6/12/24/36 months | M | 29 | N | 99 | 2 | 2 | 9.57 | no | 2 | 1  (36) |
| HSCT24 | Yes  0/3/6/12/24/36 months | Yes  0/6/12/24/36 months | M | 33 | N | 103 | 1 | 2 | 8.76 | no | 4.5 | 1  (36) |
| HSCT25 | Yes  0/3/6/12/24/36 months | Yes  0/6/12/24/36 months | M | 28 | Y | 73 | 2 | 1 | 9.98 | no | 2 | 2  (36) |
| HSCT26 | Yes  0/3/6/12/24 months  *last follow up 24 months | Yes  0/6/12/24 months | F | 37 | N | 17 | 5 | 6 | 12.42 | no | 6.5 | 4.5  (24) |
| HSCT27 | Yes  0/3/6/12/24 months  *last follow up 24 months | Insufficient sample | M | 31 | N/A | 79 | 3 | 2 | 11.12 | no | 3 | 2  (24) |
| HSCT28 | Yes  0/3/6/12/24 months  *last follow up 24 months | Yes  0/6/12/24 months | M | 44 | Y | 124 | 1 | 4 | 7.14 | Sensory disturbance involving right leg 11 months post-AHSCT. Didn’t restart DMT. | 3.5 | 4  (24) |
| HSCT30 | Yes  0/3/6/12/24 months  *last follow up 24 months | Yes  0/6/12/24 months | M | 31 | N | 19 | 2 | 5 | 14.00 | no | 2.5 | 2.5  (24) |
| NTZ01 | no | Yes  0 and 24 months | M | 41 | N | 69 | 0 (on NTZ) | 0 (on NTZ) | n/a | no | 2.5 | 2.5  (24) |
| NTZ02 | no | Yes  0 and 24 months | F | 27 | Y | 47 | 0 (on NTZ) | 0 (on NTZ) | n/a | no | 2 | 2  (24) |
| NTZ03 | no | Yes  0 and 24 months | F | 33 | Y | 56 | 0 (on NTZ) | 0 (on NTZ) | n/a | no | 2.5 | 2  (24) |

Supplementary Table 7. HLA typing

|  | HLA Class I | HLA Class II |
| --- | --- | --- |
| HSCT04 | A01:01, 01:01  B08:01, 08:01  C07:01, 07:01 | DRB1*03:01, 03:01  DPB1*01:01, 01:01  DPA1*02:01, 02:01  DQB1*02:01; 02:01  DQA1*05:01; 05:01 |
| HSCT06 | A02:01, 25:01  B44:03, 51:01  C01:02, 14:03 | DRB1*04:05; 11:01  DPB1*02:01, 04:01  DPA1*01:03, 01:03  DQB1*03:01, 03:02  DQA1*03:02, 05:05 |
| HSCT09 | A02:01, 01:01  B07:02, 08:01  C02:02, 07:01 | DRB1*04:01, 15:01  DPB1*01:01, 01:01  DQB1*02:01, 06:09  DQA1*05:01, 03:02 |
| HSCT10 | A02:01, 02:01  B07:02, 44:02  C02:02, 07:02 | DRB1*08:01, 13:01  DPB1*04:01, 04:01  DPA1*01:03, 01:03  DQB1*04:02, 06:03  DQA1*01:03, 04:01 |
| HSCT11 | A02:01, 25:01  B15:01, 18:01  C03:04, 12:03 | DRB1*07:01, 15:01  DPB1*04:02, 23:01  DPA1*01:03, 01:03  DQB1*02:02, 06:02  DQA1*01:02, 02:01 |
| HSCT13 | A02:01, 24:02  B40:01, 40:02  C03:04, 14:02 | DRB1*15:01, 04:03  DPB1*02:01, 02:01  DQB1*03:02, 06:02  DQA1*01:02, 03:01 |
| HSCT16 | A03:01, 68:01  B14:02, 14:02  C08:02, 08:02 | DRB1*13:02, 13:02  DPB1*05:01, 05:01  DPA1*02:01, 02:01  DQB1*06:09, 06:09  DQA1*01:02, 01:02 |
| HSCT22 | A01:01, 01:01  B08:01, 08:01  C07:01, 07:01 | DRB1*03:01, 03:01  DPB1*01:01, 01:01  DQB1*02:01, 02:01  DQA1*05:01, 05:01 |
| HSCT24 | A01:01, 03:01  B08:01, 13:02  C06:02, 07:02 | DRB1*04:01, 03:01  DPB1*07:01, 02:01  DQB1*02:01, 06:02  DQA1*02:02, 02:02 |
| HSCT25 | A03:01, 11:01  B18:01, 49:01  C07:01, 07:02 | DRB1*11:04, 15:01  DPB1*04:01, 04:02  DPA1*01:03, 01:03  DQB1*03:01, 05:01  DQA1*01:02, 05:05 |
| HSCT26 | A01:01, 32:01  B08:01, 08:01  C07:01, 07:01 | DRB1*03:01, 03:01  DPB1*01:01, 02:01  DQB1*02:01, 02:01  DQA1*05:01, 05:01 |
| HSCT28 | A01:01, 02:01  B08:01, 13:02  C06:02, 07:01 | DRB1*03:01, 15:01  DPB1*03:01, 04:02  DQB1*02:01, 06:02  DQA1*01:02, 05:01 |
| HSCT30 | A02:01, 02:01  B40:01, 40:01  C03:04, 03:03 | DRB1*13:01, 13:02  DPB1*04:01, 04:02  DPA1*01:03, 01:03  DQB1*06:03, 06:04  DQA1*01:02, 01:03 |
| NTZ01 | A01:01, 11:01  B08:01, 14:02  C06:02, 07:01 | DRB1*13:02, 13:02  DPB1*05:01, 05:01  DPA1*01:03, 02:01  DQB1*06:09, 06:09  DQA1*01:02, 05:01 |
| NTZ02 | A01:02, 25:01  B08:01, 40:02  C06:07, 03:01 | DRB1*15:01, 04:03  DPB1*04:01, 05:01  DQB1*06:03, 03:02  DQA1*01:02, 01:01 |
| NTZ03 | A03:01, 02:01  B18:01, 08:01  C07:01, 08:02 | DRB1*15:01, 04:01  DPB1*04:01, 06:09  DQB1*03:01, 03:01  DQA1*01:01, 02:01 |

Supplementary Figure 1. Gating strategy for the T cell reconstitution flow cytometry panel.

Final populations of interest are defined in bold and underlined. (A) Live CD3+ lymphocytes are gated for CD4+ and CD8+. (B) Both populations are then gated for ‘naïve-like’, ‘central memory’ (population of interest), ‘effector memory - like’ and ‘terminal effector - like’. (C) In both CD4+ and CD8+ subsets ‘naïve-like’ cells are then gated for CD27, for which they are all positive, and then CD95. Naïve cells (population of interest) are then defined to be CD95 negative. This population is further assessed for CD31, to determine recent thymic emigrants (CD31+, population of interest). (D) ‘Effector memory-like’ CD4+ and CD8+ cells are assessed for CD27. CD27+ cells are termed ‘transitional memory’ cells (population of interest) and CD27- cells are termed ‘effector memory’ cells (population of interest). (E) ‘Terminal effector-like’ cells are assessed for CD27. The CD27- population is termed ‘terminal effector memory’ cells (population of interest).

Supplementary Figure 2. Gating strategy for the FACS panel.

Populations of interest are defined in red. Live CD3+ lymphocytes are gated for CD4+ and CD8+. Both populations are then gated for ‘naïve – CD45RA’ and ‘memory – CD45RO’ subset’.


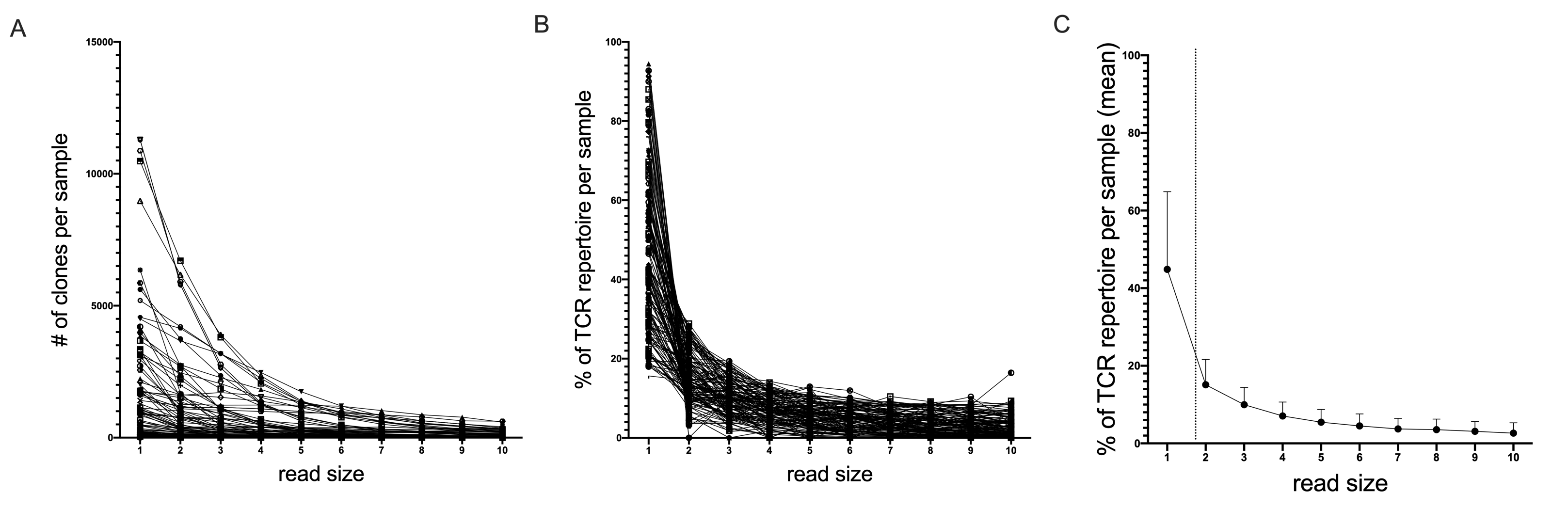


Supplementary Figure 3. Restricting the definition of a public clone. In order to analyse changes in public TCR profiles post-AHSCT a clone was defined as ‘public’ if it was identified in the TCR repertoire of 2 or more individuals at the same time point and lymphocyte subset. In order to stringently exclude erroneous reads, clonotypes were plotted against read size for all samples to determine the mean fraction of the TCR repertoire represented by read sizes 1 – 10 in order to identify a ‘true’ clone without compromising sequencing depth. For consistency in analysis of clone sharing across individuals and the restriction in patient numbers beyond 24 months, the 36-month samples were restricted from this analysis. (a) For each sample (n = 276) the number of clones [y] was plotted against a read size [x]. Only read sizes of 1 (singletons) – 10 are depicted within the graph. (b) The number of clones was then normalised to percentage of the TCR repertoire [y] in order to account for variations in the number of lymphocytes per sample. (c) The mean percentage of the TCR repertoire made up from different read sizes; singletons – 10 was then plotted to depict a ‘break point’ for defining a clone. Consistent with convention around TCR sequencing, singletons were subsequently excluded from the analysis.

Supplementary Figure 4. TCR profiles of natalizumab (NTZ)-treated patients. Percentage of the dominant (top 100) baseline (a) CD4+CD45RO+ and (b) CD8+CD45RO+ clones in NTZ03 that were detected on repeat sampling after 24 months of ongoing treatment with natalizumab. (c) Percentage of the dominant (top 100) baseline clonotypes detected at 24 months for the cohort as a whole. (d) Stable entropy in CD4+ and CD8+ CD45RO+ populations between baseline and 24 months in three NTZ treated patients. (e) Percentage of the naïve repertoire from baseline detected at 24 months.

Supplementary Figure 5. TCR specificity of the memory repertoire before and after AHSCT. The average proportion of the repertoire that could be annotated to an HLA-matched reported epitope is listed above each pie chart. The proportion of the annotated repertoire is subsequently listed below each graph, for CMV, EBV, influenza, human antigen (homosapien) and other. Values given are averages (mean) across the population sequenced. Given the variability in HLA types of patients, reporting bias in the VDJdb and TCR promiscuity no statistically significant changes in virus specific T cells over the post-transplant period could be reported.

Supplementary Figure 6. Dominant public clones pre- and post-AHSCT. The size (read size) of (A) CD4+CD45RO+ (top 100 of 181) and (B) CD8+CD45RO+ public clones (total = 48) before and 24 months after AHSCT. Read size at baseline (left hand side) and 24 months (right hand side) is depicted along the x-axis. Clones specific for virus have been coded - CMV (dark green) and EBV (orange). The V gene – J gene – CDR3 amino acid sequence of each clone is listed along the y-axis. Where multiple clonotypes (by nucleotide sequence) have contributed to a clone these have been denoted by a break in the horizontal bar to represent the read size of each nucleotide sequence.
